# Syk-Mediated Translocation of PI3Kδ to the Leading Edge Controls Lamellipodium Formation and Migration of Leukocytes

**DOI:** 10.1371/journal.pone.0001132

**Published:** 2007-11-07

**Authors:** Jürgen Schymeinsky, Cornelia Then, Anca Sindrilaru, Ronald Gerstl, Zoltán Jakus, Victor L. J. Tybulewicz, Karin Scharffetter-Kochanek, Barbara Walzog

**Affiliations:** 1 Department of Physiology, Ludwig-Maximilians-University Munich, Munich, Germany; 2 Department of Dermatology and Allergic Diseases, University of Ulm, Ulm, Germany; 3 Department of Physiology, Semmelweis University School of Medicine, Budapest, Hungary; 4 Division of Immune Cell Biology, National Institute for Medical Research, London, United Kingdom; University of Cambridge, United Kingdom

## Abstract

The non-receptor tyrosine kinase Syk is mainly expressed in the hematopoietic system and plays an essential role in β_2_ integrin-mediated leukocyte activation. To elucidate the signaling pathway downstream of Syk during β_2_ integrin (CD11/CD18)-mediated migration and extravasation of polymorphonuclear neutrophils (PMN), we generated neutrophil-like differentiated HL-60 (dHL-60) cells expressing a fluorescently tagged Syk mutant lacking the tyrosine residue at the position 323 (Syk-Tyr^323^) that is known to be required for the binding of the regulatory subunit p85 of the phosphatidylinositol 3-kinase (PI3K) class I_A_. Syk-Tyr^323^ was found to be critical for the enrichment of the catalytic subunit p110δ of PI3K class I_A_ as well as for the generation of PI3K products at the leading edge of the majority of polarized cells. In accordance, the translocation of PI3K p110δ to the leading edge was diminished in Syk deficient murine PMN. Moreover, the expression of EGFP-Syk Y323F interfered with proper cell polarization and it impaired efficient migration of dHL-60 cells. In agreement with a major role of β_2_ integrins in the recruitment of phagocytic cells to sites of lesion, mice with a Syk-deficient hematopoietic system demonstrated impaired PMN infiltration into the wounded tissue that was associated with prolonged cutaneous wound healing. These data imply a novel role of Syk via PI3K p110δ signaling for β_2_ integrin-mediated migration which is a prerequisite for efficient PMN recruitment in vivo.

## Introduction

The efficient recruitment of polymorphonuclear neutrophils (PMN) to sites of lesion is critical for host defense, inflammation and tissue repair [1], [2]. An essential step during this process is chemotactic migration which requires the presence of chemoattractants and depends on adhesive cell-matrix interactions that are mainly mediated by the leukocyte adhesion molecules of the integrin family [3], [4]. Especially, β_2_ integrins (CD11/CD18) are critically involved in firm adhesion and migration of PMN [4], [5]. The β_2_ integrins are heterodimers consisting of a common β-subunit (CD18) which is non-covalently associated with one of the four known α-subunits (CD11) and are named accordingly: CD11a/CD18 (αLβ_2_, LFA-1), CD11b/CD18 (αMβ_2_, Mac-1, CR3), CD11c/CD18 (αXβ_2_, gp150/95, CR4) or CD11d/CD18 (αDβ_2_) [5]. The pivotal role of CD18 during the acute inflammatory response becomes obvious in patients suffering from leukocyte adhesion deficiency type-1 (LAD-1) where a defective CD18 expression is the cause of impaired wound healing and recurrent bacterial infections due to a recruitment defect of PMN [4]. Accordingly, a recent in vivo study demonstrated an impaired adhesion of LFA-1^−/−^ PMN to the wall of postcapillary venules of the murine cremaster muscle upon stimulation with macrophage inflammatory protein-2 (Mip-2) and a decreased intraluminal crawling of Mac-1^-/-^ PNM accompanied by a delayed emigration through nonoptimal sites [6]. Downstream signaling of CD18 via specific Immunoreceptor Tyrosine-based Activation Motif (ITAM)-bearing adapter proteins (i.e. DAP12 and the Fc receptor (FcR) γ-chain) leads to the activation of the non-receptor tyrosine kinase Syk [7]–[9] which has been shown to be critical for migration of neutrophil-like differentiated HL-60 cells (dHL-60) and primary murine PMN [10], [11]. Syk has several interacting signaling partners e.g. Vav, the phophoinositide-specific phospholipase C (PLC) isozymes PLCγ-1 and PLCγ-2, and Cbl which are known to exert a functional impact on different leukocyte functions including cell migration [10]–[17]. Recently, a direct interaction between Syk and the regulatory subunit p85 of the phosphatidylinositol 3-kinases (PI3K) class I_A_ has been demonstrated [13], [18].

PI3Ks are grouped into three classes, I, II and III, according to their substrate preference and structural homology [19]. Class I PI3Ks, which produce the secondary messenger phosphatidylinositol-(3,4,5)-trisphosphate (PIP3), are divided into two subgroups, I_A_ and I_B_. Class I_A_ PI3Ks are tightly bound heterodimers that consists of a p50–55/p85 regulatory subunit and one of the three p110 catalytic subunits, p110α, p110β or p110δ. The three isoforms of the p85 regulatory subunit (p85α with its splice products p55α and p50α, p85β and p55γ) mediate the activation of class I_A_ PI3K by binding to receptor tyrosine kinases and adaptor molecules. This binding relieves the basal inhibition of p110 by p85 and recruits the p85–p110 heterodimer to its substrate at the plasma membrane. Class I_B_ PI3Ks are dimers consisting of the catalytic subunit p110γ and one of the regulatory subunits p101 or p84 (p87^PIKAP^) [19], [20]. The PI3K signaling pathway is critically involved in the control of PMN motility and chemotaxis [3] and β_2_ integrins are capable of activating PI3K in PMN [21]. In these cells, chemoattractants can activate both the class I_A_ PI3K as well as the class I_B_ PI3K, i.e. p110γ which is regulated by G-protein coupled receptor signaling [19], [22]. Furthermore, a relevance of Syk in the activation of PI3K has been described for T cell and B cell receptor-mediated signaling and in PMN after the stimulation with monosodium urate crystals [23]–[25].

In the present study, we unraveled the physiological role of Syk-mediated translocation and activation of PI3K p110δ upon adhesion and polarization of leukocytes in the presence of the β_2_ integrin ligand fibrinogen. Cell polarization and migration of dHL-60 cells expressing fluorescently tagged wildtype Syk and a Syk mutant lacking the binding site for the p85 regulatory subunit of PI3K Class I_A_ (EGFP-Syk Y323F, mCherry-Syk Y323F) were analyzed in vitro by confocal microscopy and time-lapse video microscopy. To address the in vivo relevance of Syk-mediated signaling for PMN recruitment to sites of lesion, we performed a wound healing assay using wildtype mice reconstituted with a Syk deficient hematopoietic system.

## Materials and Methods

### Reagents and antibodies

BSA, human fibrinogen, fMLP and Percoll^TM^ were obtained from Sigma, Deisenhofen, Germany. Piceatannol was obtained from Calbiochem, La Jolla, CA, USA. We purchased RPMI 1640 medium, FCS, penicillin, streptomycin and PBS from Biochrom, Berlin, Germany. IMDM including L-Glutamine and 25 mM HEPES was purchased from Invitrogen, Karlsruhe, Germany. Antibodies to Syk (clone 4D10, IgG_2a_), p110δ (H-219, sc-7176), p110γ (H-199, sc-7177), Cbl (C-15, sc-170), PLCγ-1 (2R1, sc-58408), PLCγ-2 (B-10, sc-5283), or Dock2 (E-15, sc-50921) were obtained from Santa Cruz, Santa Cruz, CA, USA. The monoclonal anti-CD18 antibody (clone MHM23) was purchased from DakoCytomation, Glostrup, Denmark. Alexa fluorochrome-conjugated secondary antibodies and phalloidin were purchased from Invitrogen, Karlsruhe, Germany.

### cDNA contructs

The EGFP-tagged Syk Y323F mutant was obtained from the non-mutated EGFP-Syk plasmid as described previously [10]. The used DNA oligonucleotides 5′-TGTCATTCAATCCGTTT-GAGCCAGAACTTGG-3′ and its complement reverse form were obtained from Metabion, Martinsried, Germany. The introduction of the expected mutation was confirmed by automated DNA sequencing (Medigenomix, Martinsried, Germany). For the expression of red fluorescent fusion proteins the coding region of EGFP of the EGFP-C1 expressing vector (Clontech, BD New York, USA) was replaced with a PCR-DNA Fragment of the mCherry cDNA flanked with a NheI and a BglII restriction site (DNA oligonucleotides: forward 5′-GCTAGCGCCACCATGGTGAGC-AAGGGCGAG-3′, and reverse 5′-CTGACAGATCTCTTGTACAGCTCGTCCATGC-3′). The mCherry cDNA was a generous gift of Dr. Roger Y. Tsien, Dept. of Chemistry and Biochemistry, University of California at San Diego, La Jolla, CA, USA [26]. The coding region of wildtype Syk or Syk Y323F was cloned into the *EcoR*I/*Kpn*I site of the newly synthesized mCherry-C1 vector.

### Cell culture and electroporation of dHL-60 cells

The human myeloid HL-60 cell line (ACC 3) was obtained from the German Resource Centre for Biological Material (Braunschweig, Germany). The HL-60 cell line stably expressing PH-AKT-GFP was kindly provided by Dr. H. R. Bourne, UCSF, CA, USA [27]. PMN-like differentiation of HL-60 cells and the electroporation protocol was described previously [10]. The generation of the HL-60 cell line stably expressing a Syk specific short hairpin RNA which led to a reduced Syk expression via the generation of short interfering RNA (Syk-siRNA) was generated as described [10].

### Isolation of murine bone marrow PMN

Murine bone marrow PMN were isolated as described previously [10]. Briefly, the bone marrow from femurs and tibias was isolated and subsequently loaded on top of a discontinuous Percoll™ gradient (52%/64%/72%) and centrifuged at 1000 g for 30 min. PMN were harvested from the 64%/72% interface and cultivated for 24 h in RPMI 1640 medium supplemented with 20% of WEHI-3B-conditioned medium. PMN viability was >95% as assessed by the trypan blue exclusion test, purity was >98% as analyzed by microscopy using Hemacolor™ staining (Merck, Darmstadt, Germany).

### Functional assays and microscopy

Adhesion and migration assays and time-lapse video microscopy was performed as described previously. Polarized dHL-60 cells were prepared for staining and used for confocal microscopy as described [10].

### Wound-healing model

The wound healing assay was performed as described [28]. Briefly, after anaesthetizing the mice, excisional wounds were punched at four sites in the middle of the dorsum using 5-mm biopsy stamps (STIEFEL, Offenbach, Germany). Each wound region was digitally photographed at indicated time points, and areas were calculated using photoshop software (Adobe Systems, San Jose, CA, USA). For immunohistochemistry wound granulation tissues were fixed in 4% neutral buffered formalin and embedded in paraffin. Neutrophils were detected using an anti-Gr-1 antibody (clone RB6-8C5, BD Pharmingen, Heidelberg, Germany) as described [28]. Slides were visualized using a Zeiss Axiophot microscope (Carl Zeiss Inc., Oberkochen, Germany) and pictures were acquired with a digital color camera and corresponding software (Axiocam®, Zeiss). Mice carrying the Syk^tm1Tyb^ (referred to as Syk^−^) mutation were maintained as Syk^+/−^ heterozygotes on the C57BL/6 genetic background [29]. The generation of mice with a Syk^−/−^ hematopoietic system was performed as described previously [10].

### Statistical analysis

Data shown represent means±SD. Statistical significance was determined using a two-tailed Student's *t*-test or the Mann-Whitney U-test, in case of non-normal distributions. p<0.05 was considered statistically significant.

## Results

### Syk-Tyr^323^ is required for the consolidation of the leading edge and for efficient migration of dHL-60 cells

**Figure 1:**
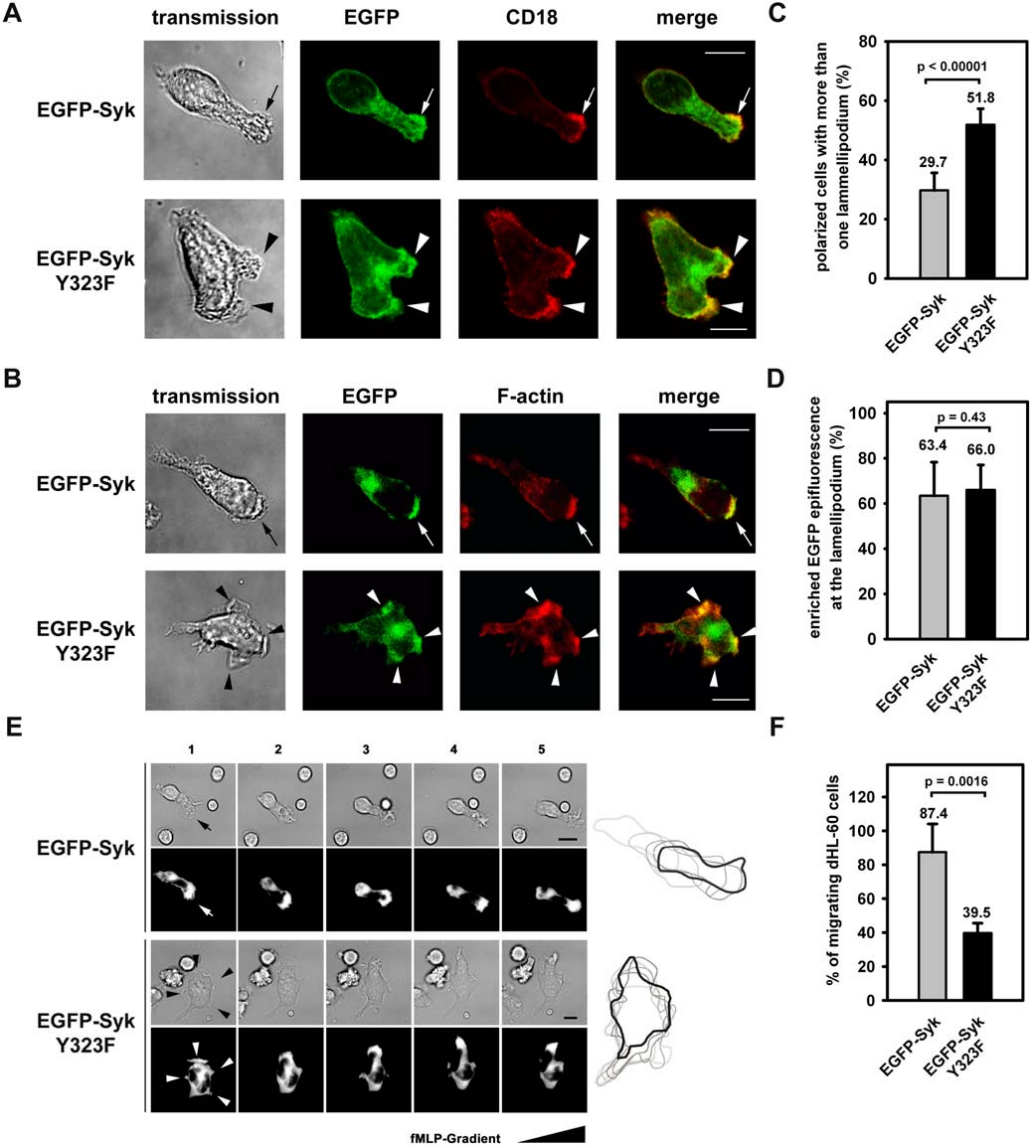
Syk-Tyr^323^ was required for the consolidation of the leading edge during CD18-mediated migration of dHL-60 cells. (A,B) Confocal microscopy images of dHL-60 cells expressing EGFP-Syk or EGFP-Syk Y323F stained for CD18 (A) or F-actin (B). Upon stimulation by 100 nM fMLP on immobilized fibrinogen, all transfectants polarized and formed lamellipodia (black arrows and arrow heads), which were characterized by an enrichment of CD18, F-actin, EGFP-Syk, or EGFP-Syk Y323 (white arrows and arrow heads). However, the expression of the Syk mutant led to the formation of multiple lamellipodia (arrow heads). Pictures are representative of at least three independent experiments. Quantitative analysis of microscopic images of fixed dHL-60 cells showing the percentage of cells with (C) excessive lamellipodium formation and (D) enhanced EGFP epifluorescence at the lamellipodium (n>300 cells each, taken from at least three independent experiments). (E) Time-lapse microscopy of migrating dHL-60 transfectants on immobilized fibrinogen in response to a gradient of 10 nM fMLP (▴). Images 1–5 were recorded at intervals of 30 s. Control transfectants migrated efficiently in contrast to Syk Y323F mutants which formed multiple, unstable lamellipodia and showed impaired migration. The outlines of the cells at intervals of 30 s were transposed in rising grayscales to visualize cell movement. (F) Percentage of migrating dHL-60 transfectants (n = 63 EGFP-Syk, n = 67 EGFP-Syk Y323F) from at least four independent experiments. Data represent means±SD, bar = 10 µm.

To characterize the CD18-mediated signaling pathway downstream of Syk during PMN migration and infiltration, we generated dHL-60 cells transiently expressing an EGFP-tagged Syk mutant (EGFP-Syk Y323F), where the major binding site of the regulatory subunit p85 of the PI3K class I_A_ was removed [13], [18]. As expected, EGFP-Syk expressing cells polarized upon stimulation by 100 nM fMLP on immobilized fibrinogen and the majority of these cells formed one lamellipodium that was characterized by an enrichment of F-actin and CD18. However, 29.7% of the cells formed more than one lamellipodium (Figure 1A and C). In contrast, 51.8% of the EGFP-Syk Y323F expressing cells showed an excessive lamellipodium formation, whereas CD18 was still enriched at the leading edge. Furthermore, dHL-60 cells expressing EGFP-Syk enriched Syk at the lamellipodium (63.4%) which is in accordance with our previous findings (Figure 1B and D) [10]. Similarly, 66.0% of the Syk Y323F mutants showed an enhanced EGFP fluorescence at the lamellipodium indicating that Syk-Tyr^323^ was not required for the translocation of Syk but involved in the consolidation of the leading edge. As the establishment of ordered cell polarity is a prerequisite for efficient cell migration, we performed migration assays using dHL-60 cells expressing EGFP-Syk or EGFP-Syk Y323F. Whereas 87.4% of the EGFP-Syk transfectants performed efficient migration on immobilized fibrinogen along a gradient of 10 nM fMLP (Figure 1E and F), this percentage was significantly reduced to 39.5% in cells expressing the Syk mutant which again formed multiple and unstable lamellipodia (Please see also Movie S1 and Movie S2 presented as supplemental information). These results indicate that Syk-Tyr^323^ supported the maintenance of cell polarity, a prerequisite for efficient cell migration suggesting that the PI3K class I_A_ may act downstream of Syk.

### The enrichment of PI3K p110δ at the leading edge of dHL-60 cells depends on Syk-Tyr^323^

**Figure 2:**
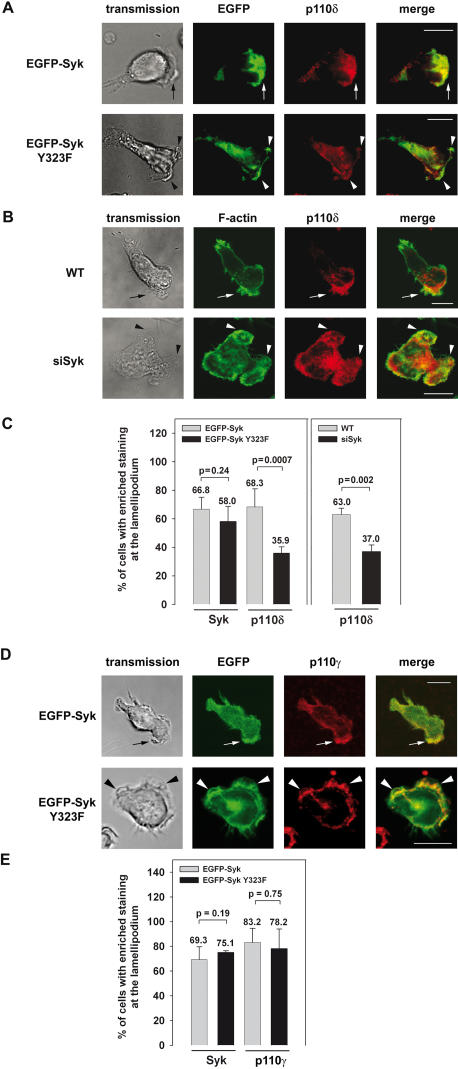
Syk was required for the translocation of PI3K p110δ but not of p110γ to the leading edge. Microscopy images of dHL-60 cells expressing EGFP-Syk or EGFP-Syk Y323F (A,D) or stably expressing a Syk-siRNA (siSyk) (B). Upon stimulation by 100 nM fMLP on immobilized fibrinogen, PI3K p110δ was enriched at the lamellipodium (arrow) of (A) EGFP-Syk transfectants and (B) dHL-60 control cells (WT). In contrast, dHL-60 cells expressing EGFP-Syk Y323F or Syk-siRNA (siSyk) showed an impaired enrichment of PI3K p110δ staining at the leading edge (arrow heads). (C) Quantitative analysis of subcellular Syk and PI3K p110δ distribution using microscopic images of fixed dHL-60 cells from at least three independent experiments. (n = 67 EGFP-Syk, n = 88 EGFP-Syk Y323F; n = 210 WT, n = 138 Syk-siRNA). (D) PI3K p110γ was translocated to the leading edge in EGFP-Syk (arrow) as well as in EGFP-Syk Y323F transfectants (arrow heads). (C) Quantitative analysis (n = 48 EGFP-Syk, n = 49 EGFP-Syk Y323F). Data represent means±SD, bar = 10 µm.

The class I_A_ PI3K p110δ is predominantly expressed in leukocytes and it is known to play an essential role in PMN chemotaxis [30]–[32]. As the precise temporal and spatial distribution of activated signal transducers is critical for the control of cell functions such as migration [3], we analyzed the subcellular distribution of PI3K p110δ in polarized EGFP-Syk Y323F transfectants and in dHL-60 EGFP-Syk expressing control cells. Upon induction of cell polarization on immobilized fibrinogen with a uniform concentration of 100 nM fMLP, PI3K p110δ staining was enriched at the lamellipodium in 68.3% of the control cells (Figure 2A and C). In contrast, the enrichment of PI3K p110δ at the leading edge was significantly impaired to 35.9% in the Syk mutant although an enhanced EGFP-Syk Y323 epifluorescence similar to the control transfectants was still detectable at the lamellipodium (66.8% and 58.0%, respectively). The finding that intact Syk was involved in the efficient translocation of p110δ to the leading edge was confirmed using a previously described HL-60 cell clone that downregulates Syk protein expression by stably producing a Syk specific short-interfering RNA (Syk-siRNA) [10]. The Syk-siRNA dHL-60 cells formed multiple lamellipodia and showed an impaired enrichment of PI3K p110δ at the leading edge in 37.0% of the cells compared to 63.0% of the wildtype cells (Figure 2B and C). These findings confirm that Syk was required for the observed asymmetric distribution of PI3K p110δ within the cell. Recent studies have demonstrated a critical role for PI3K p110γ in the control of PMN migration [33], [34]. To ask whether Syk-Tyr^323^ is required for the translocation of PI3K p110γ during β_2_ integrin-mediated adhesion and polarization in leukocytes, we analyzed the subcellular localization of PI3K p110γ in EGFP-Syk Y323F or EGFP-Syk expressing dHL-60 cells. The majority of the polarized EGFP-Syk and EGFP-Syk Y323F expressing cells showed not only an enrichment of EGFP epifluorescence (69.3% and 75.1%, respectively) but also an accumulation of PI3K p110γ staining (83.2% and 78.2%) at the lamellipodium forming site indicating that the translocation of the Class I_B_ PI3K p100γ was independent of Syk-Tyr^323^ (Figure 2D and E).

**Figure 3:**
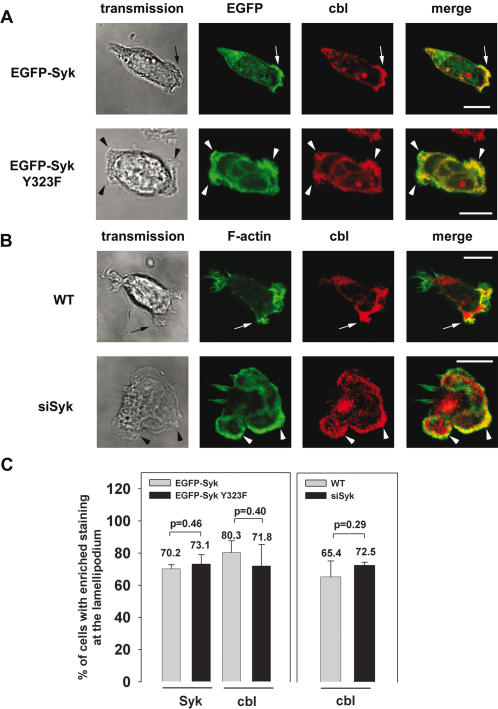
The enrichment of Cbl at the lamellipodium was not dependent on Syk-Tyr^323^. Confocal microscopy images of dHL-60 cells (A) expressing EGFP-Syk or EGFP-Syk Y323F or (B) stably expressing a Syk-siRNA (siSyk). Upon stimulation by 100 nM fMLP on immobilized fibrinogen, Cbl was enriched at the lamellipodium of (A) EGFP-Syk transfectants and (B) dHL-60 control cells (arrow and arrow heads). A similar result was obtained in dHL-60 cells expressing EGFP-Syk Y323F or siSyk. (C) Quantitative analysis of subcellular Syk and Cbl distribution using microscopic images of fixed dHL-60 cells taken from three independent experiments (n = 77 EGFP-Syk, n = 78 EGFP-Syk Y323F; n = 177 WT, n = 105 Syk-siRNA). Data represent means±SD. Bar = 10 µm.

Since it is known that the adaptor protein Cbl binds to Syk-Tyr^323^ [35] and expression and tyrosine phosphorylation of Cbl regulates macrophage chemokinetic and chemotactic movement [17], we analyzed the EGFP-Syk transfectants and Syk-siRNA expressing cells for the subcellular distribution of Cbl (Figure 3). The majority of EGFP-Syk expressing control cells as well as EGFP-Syk Y323F transfectants showed enrichment of Syk (70.2% and 73.1%) and of Cbl (80.3% and 71.8%) at the leading edge. Moreover, downregulation of Syk by RNAi technique did not affect the translocation of Cbl to the leading edge indicating that Syk-Tyr^323^ as well as a certain threshold of Syk protein was not required for the enrichment of Cbl at the lamellipodium forming site. Thus, Syk-Tyr^323^ is specifically required for accumulation of PI3K p110δ but dispensable for the enrichment of Cbl at the leading edge. In a previous study, we have shown that Syk-Tyr^348^ was indispensable for the recruitment of the guanine nucleotide exchange factor Vav to the leading edge and the control of leukocyte migration [10]. However, this process seems to be independent of Syk-Tyr^323^-mediated signaling, since the enrichment of Vav at the leading edge was not affected in the Syk-Tyr^323^ mutants (supporting information Figure S1 A and E). To investigate whether Syk-Tyr^323^ has an impact on the translocation of other potential downstream signaling components of Syk, we investigated the subcellular localization of dedicator of cytokinesis 2 (Dock2), PLCγ-1 and PLCγ-2 in dHL-60 cells expressing wildtype Syk and the Syk mutant. Dock2, an activator of Rac and a member of the CDM family proteins (*Caenorhabditis elegans* CDE-5, mammalian Dock180 and *Drosophila melanogaster* Myoblast City), has been recently described to be required for the migration and polarization during PMN chemotaxis and its translocation to the leading edge has been reported to depend on PI3K activity [36]. However, the translocation of all these proteins to the leading edge did not depend on Syk-Tyr^323^ (supporting information Figure S1 B–E). Thus, Syk-Tyr^323^ seems to be specifically required for the enrichment of PI3K p110δ to the leading edge of polarized leukocytes.

### The spatio-temporal control of PI3K activity is mediated by Syk-Tyr^323^

**Figure 4:**
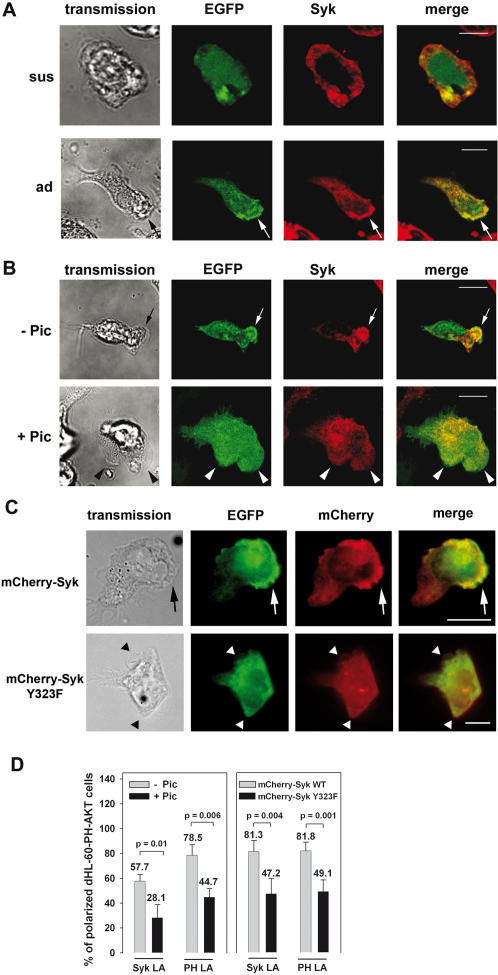
Syk activity was required for the enrichment of PI3K products at the leading edge. Microscopy images of PH-AKT-GFP dHL-60 cells in suspension (sus) or after induction of adhesion (ad) to immobilized fibrinogen in the presence of 0.2 mM Mn^2+^ (A), or adhered and polarized after the induction with 100 nM fMLP (B, C). PH-AKT-GFP dHL-60 cells were treated with 30 µM piceatannol (+Pic) or vehicle for control (-Pic) (B), or expressed mCherry-Syk or mCherry-Syk Y323F (C). PH-AKT-GFP dHL-60 cells stimulated in suspension did not translocate Syk or PH-AKT-GFP (EGFP) to the membrane. In contrast, adherent cells showed an enriched Syk staining, or mCherry-Syk and PH-AKT-GFP epifluorescence at the lamellipodium forming site (arrow). Inhibition of Syk or the expression of mCherry-Syk Y323F increased the population of cells with a homogenous distribution of Syk and a diminished concentration of PH-AKT-GFP at the lamellipodium forming site (arrow head). Pictures are representative for at least three independent experiments. (D) Quantitative analysis of the subcellular localization of Syk, mCherry-Syk or mCherry-Syk Y323F (Syk LA) or PH-AKT-GFP epifluorescence (PH LA) at the lamellipodium in PH-AKT-GFP dHL-60 cells taken from at least three experiments (n = 137−Pic, n = 157+Pic; n = 113 mCherry-Syk, n = 99 mCherry-Syk Y323F). Data represent means±SD. Bar = 10 µm.

To study the functional relevance of the impaired translocation of PI3K p110δ to the leading edge, we measured PI3K activity indirectly using the PH-AKT-GFP HL-60 cell line. This cell line constitutively expresses the pleckstrin homologous (PH) domain of the AKT kinase fused to an EGFP tag that functions as a biosensor for PI3K activity by binding to PI3K products in live cells [27]. In suspended PH-AKT-GFP dHL-60 cells, Syk and PH-AKT epifluorescence were homogenously distributed after stimulation (Figure 4A). Upon induction of cell adhesion on immobilized fibrinogen with 0.2 mM Mn^2+^ or 100 nM fMLP, the biosensor PH-AKT-GFP was enriched at the lamellipodium of dHL-60 cells indicating a role of β_2_ integrin-mediated adhesion and signaling for the subcellular generation of PI3K products. To analyze the role of Syk for the activation of PI3K, we used the Syk specific inhibitor piceatannol (Figure 4B). Upon inhibition of Syk, the cells formed multiple lamellipodia in response to fMLP-stimulation and showed a decreased enrichment of PH-AKT-GFP epifluorescence at the lamellipodium (from 78.5% of the control cells to 44.7% in the piceatannol treated cells) indicating a role of Syk for the maintenance of cell polarity by the spatio-temporal control of PI3K activity (Figure 4D). This finding is consistent with the view that the local generation of PI3K products at the leading edge provides a positive feedback signal that is required for the consolidation of the leading edge [3], [19]. To address specifically the role of Syk Tyr^323^ in the regulation of PI3K activity we analyzed PH-AKT-GFP dHL-60 cells expressing fusion proteins of wildtype Syk or the Syk Y323F mutant with the red fluorescent reporter protein mCherry (mCherry-Syk and mCherry-Syk Y323F, respectively). Similar to the experiments described above, mCherry-Syk as well as PH-Akt-GFP were enriched at the leading edge in the majority of polarized PH-Akt-GFP dHL-60 cells on immobilized fibrinogen after the stimulation with 100 nM fMLP (81.3% and 81.8%, respectively; Figure 4C and 4D). In accordance with the result obtained upon pharmacological inhibition of Syk using piceatannol the expression of mCherry-Syk Y323F led to an impaired translocation of PH-AKT-GFP to the lamellipodium in 49.1% of the dHL-60 cells. In contrast to the cells expressing EGFP-Syk Y323F alone (Figure 1), cells expressing both PH-AKT-GFP and mCherry-Syk Y323F showed an attenuated enrichment of mCherry-Syk Y323 at the leading edge which may reflect the breakdown of PI3K signaling caused by the overexpression of PH-AKT-GFP in combination with hampered PI3Kδ signaling. However, the underlying mechanism is unknown. Nevertheless, our results demonstrate that Syk-Tyr^323^ is involved in the complex spatio-temporal regulation of PI3K activity.

### The role of Syk in native PMN

**Figure 5:**
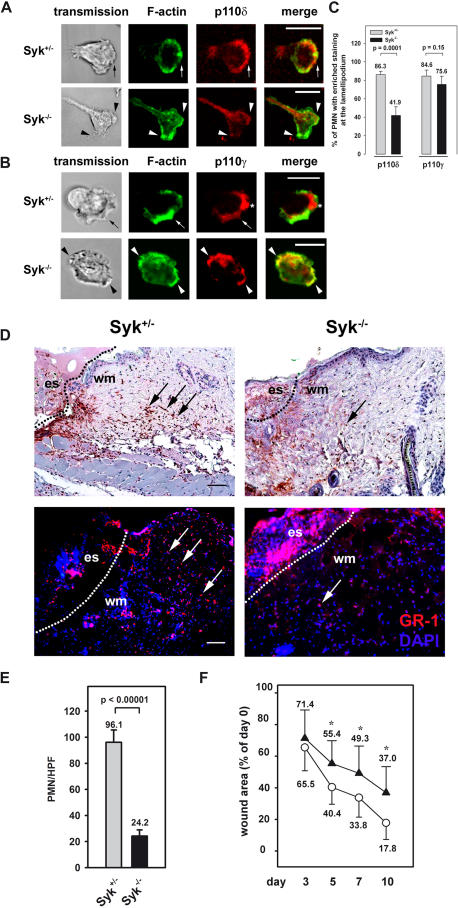
Syk was required for the enrichment of PI3K p110δ at the leading edge of murine PMN and delayed wound closure. (A,B) Confocal microscopy images of Syk-deficient (Syk^−/−^) and control (Syk^−/+^) PMN of bone marrow chimeric mice. Upon stimulation by 1 µM fMLP on immobilized fibrinogen, PI3K p110δ and PI3K p110γ were enriched at the lamellipodium (arrow) of polarized cells. In contrast, Syk^−/−^ PMN showed an impaired enrichment of PI3K p110δ staining at the leading edge (arrow heads, A) whereas PI3K p110γ was still translocated to the lamellipodium forming sites (Β). Bar = 10 µm. (C) Quantitative analysis of subcellular localization of PI3K p110δ and PI3K p110γ distribution using microscopic images of fixed murine PMN from at four independent experiments. Data represent means±SD. (D) 24 h old wound sections of wildtype mice reconstituted with a Syk^−/−^ or a Syk^+/−^ hematopoietic system for control stained with the PMN specific Gr-1 antibody (upper panel) or GR-1 antibody and DAPI (lower panel). Control sections showed substantial infiltration of Syk^+/−^ PMN (arrows) into the wounded tissue (wm: wound margin at dotted line, es: eschar). In contrast, Syk^−/−^ PMN migration into the wounded tissue was impaired. Scale bars, 100 µm. (E) Quantitative analysis of PMN numbers per HPF in the wound tissue using microscopic images. (F) Wound sizes at given time points after wounding is displayed as percentage of initial (day 0) wound area for Syk^+/−^ (○) or Syk^−/−^ (▴) bone marrow chimeric mice (100%). Data represent means±SD; n = 5 mice with four wounds per animal; p_day3_ = 0.24, *p_day5–10_<0.001.

To address the relevance of Syk for the translocation of PI3K p110δ or p100γ in native PMN, we isolated and analyzed murine PMN from the bone marrow of wildtype mice reconstituted with a Syk-deficient or a Syk heterozygous hematopoietic system for control. After the induction of cell polarization with 1 µM fMLP on immobilized fibrinogen, the majority of the control cells translocated PI3K p110δ (86.3%) and PI3K p110γ (84.6%) to the leading edge (Figure 5A, B and C). In contrast, Syk deficient PMN showed a diminished translocation of class I_A_ PI3K p110δ (41.9%) whereas class I_B_ PI3K p110γ (75.6%) was still enriched at the lamellipodium forming sites (Figure 5A, B and C). These findings are in accordance with the above described results using the dHL-60 cells and propose a selective role for Syk in the spatio-temporal control of the class I_A_ PI3K p110δ in native murine PMN. It is known that PI3K p110δ is critically involved in the recruitment of PMN into inflamed tissue [32]. To address the functional relevance of Syk for PMN infiltration and wound healing in a skin model of inflammation and repair, we produced full-thickness excisional wounds on the back of wildtype mice reconstituted with a Syk deficient or a Syk heterozygous hematopoietic system for control. PMN infiltration into the wound area was analyzed 24 h after wounding by immunostaining using the PMN specific GR-1 antibody (Figure 5A). In the wounds of mice with a Syk deficient hematopoietic system, the number of PMN (24.2±4.8 PNM per high power field) was significantly decreased compared to control mice (96.1±9.5 PMN per high power field; Figure 5B). Furthermore, wound closure was significantly delayed after 10 days in WT mice with a Syk deficient hematopoietic system to 37.0% compared to control mice were the wound size was reduced to 17.8% of the initial wound area of 100% (Figure 5C). Thus, our findings indicate a role of hematopoietic Syk for PMN infiltration into the wounded tissue and for cutaneous wound healing.

## Discussion

**Figure 6:**
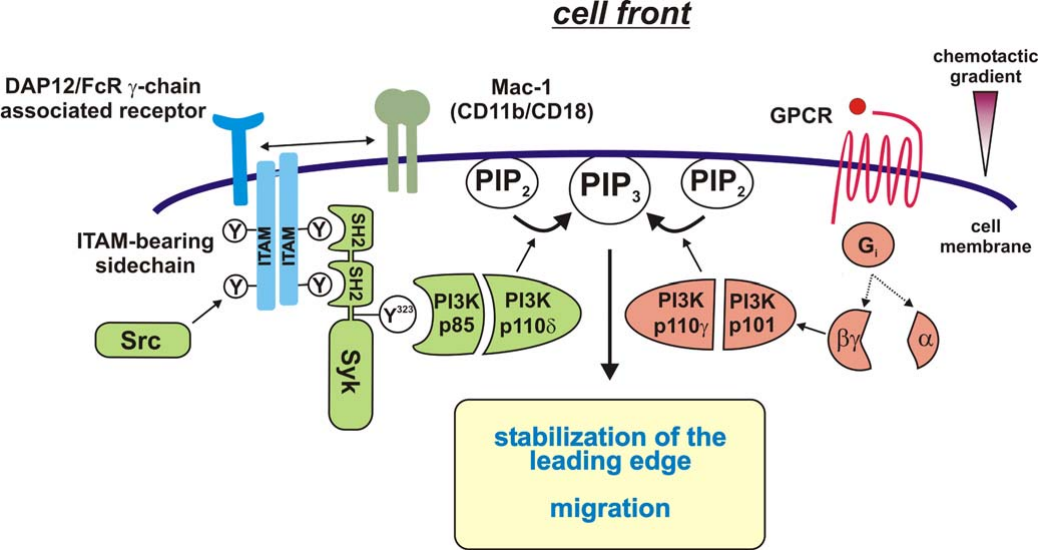
Model for localized accumulation of PI3K products at the leading edge of migrating leukocytes. Upon β_2_ integrin-mediated adhesion of PMN, the ITAM-bearing adaptor proteins DAP12 and/or the FcR γ-chain are phosphorylated by Src-family kinases. Syk binds to the phosphorylated ITAM, becomes activated and enriched at the leading edge. DAP12 and the FcR γ-chain are associated with different receptors, but it is unknown to date how these receptors interact with the β_2_ integrin Mac-1. Here, we have shown that Syk-Tyr^323^ and Syk activity were required for the recruitment of PI3K p110δ and the accumulation of PI3K products at the cell front. In contrast, the translocation of PI3K p110γ to the leading edge was independent of Syk activity and Syk-Tyr^323^. Translocation and activation of PI3K p110γ is mediated by GPCR-mediated signaling. Upon GPCR activation the α-subunit of the heterotrimeric G_i_ protein dissociates from the receptor and the βγ-subunit [22]. The βγ-complex of the G protein is known to activate PI3Kγ [19], [20], [22]. Taken together, Syk activity and Syk-Tyr^323^ mediated signaling via PI3K p110δ was required for the persistent accumulation of PI3K products at the cell front which is a prerequisite for the stabilization of the leading edge and efficient migration.

PMN are the first inflammatory cells invading the wound site after injury. Besides their defense functions against intruding microorganisms, PMN are an important source of signals that are essential for the control of their own accumulation, activation and apoptosis as well as for the attraction of macrophages and the orchestration of tissue repair including elimination of cell debris [1]. An essential step during PMN recruitment to sites of lesion are the processes of intravascular crawling and of cell migration within the tissue. PI3K signaling is known to be required for the establishment and the maintenance of cell polarity by regulating the subcellular localization and activation of downstream effectors that are essential for proper chemotaxis [3], [19]. Here, we show that Syk-Tyr^323^, the binding site for the regulatory subunit p85 of the PI3K class I_A_, was required for the enrichment of PI3K p110δ at the lamellipodium of polarized dHL-60 cells (Figure 2 and 6). This signaling step was critical for the consolidation and the stability of the leading edge and for efficient migration of dHL-60 cells on immobilized fibrinogen. This effect may be specific for class I_A_ PI3K since the translocation of PI3Kγ was not dependent on Syk-Tyr^323^. A requirement of PI3K p110δ activity in the control of chemotaxis has been demonstrated for human PMN in an under agarose assay [31] and for murine PMN in a transwell assay [32]. However, random movement seemed not to be dependent on PI3K p110δ activity at least in the human system [31]. Similarly, the inhibition of PI3K p110γ has been found to induce the formation of multiple transient lamellipodia and to inhibit migration of dHL-60 cells on immobilized fibronectin towards fMLP [37]. However, in this setting the pharmacological inhibition of PI3K p110δ by using the compound IC87114 had no effect on cell polarization and chemotaxis [37]. A reason for these controversial findings could be that the regulation of PI3K p110δ and p110γ activity may be substrate specific. This would be in accordance with a study of Ferguson et al. showing that the motility of PI3K p110γ-deficient PMN compared to control cells was not significantly reduced on glass, but it was significantly impaired on surfaces to which the cells bind less tightly, namely fibrinogen or polycarbonate [34]. However, the PMN migration assay was conducted on immobilized fibrinogen in the present study. In this model, PMN migration strongly depends on β_2_ integrins [10] and thereby reflects an inflammatory situation in vivo where PMN migrate on fibrin within the inflamed interstitial space.

Syk-Tyr^323^ has been described to be required for the binding of the phosphotyrosine-binding (PTB) domain of Cbl [35], [38]. However, our findings suggest that this interaction is not necessary for the translocation of Cbl to the leading edge. We have shown previously that the Syk substrate Cbl becomes tyrosine phosphorylated upon β_2_ integrin-mediated adhesion of human PMN suggesting a functional link between Syk and Cbl [39]. Accordingly, a negative regulatory function of Cbl in Syk-Tyr^323^-mediated signaling has been described to be required for the control of different biochemical pathways in B cells, e.g. for the activation of the transcription factor NF-AT or for B cell antigen receptor induced Ca^2+^ mobilization [35], [38], [40], and for the control of histamine release in mast cells [41]. Furthermore, Cbl was involved in the recruitment of p85 to CD28 in T cells [42]. Thus, Syk-Tyr^323^ seems not to be involved in the tranlsocation of Cbl which may not be true for the regulation of the function of Cbl.

Recently, we have reported that the redistribution of Vav depended on Syk activity and the binding site of Vav, Syk-Tyr^348^ [10]. In this study, we have shown that Syk-Tyr^323^ was not required for the translocation of Vav to the leading edge. However, effective binding of Vav, PLCγ-1 and PLCγ-2, to Syk is mediated by the phosphorylation of the Syk tyrosines Syk-Tyr^348^ and Syk-Tyr^352^ [13], [43]. Therefore, the translocation of Vav (and perhaps also PLCγ-1 and PLCγ-2) to the leading edge may be still supported by the redistribution of Syk via β_2_ integrins; a process that seems to be independent of Syk-Tyr^323^. Similar to Vav, PLCγ, can be recruited to the cell membrane via its pleckstrin homologous (PH) domain that binds the PI3K products, PI(3,4)P_2_ and/or PIP_3_ [36], [44], [45]. However, our results showed clearly that at least the activity of PI3K p110δ was not required for the enrichment of Vav and the two PLCγ isoforms at the leading edge. Since it has been described that not just the translocation but also the enzyme activity of Vav is regulated by the lipid products of the PI3K it is possible that Syk-Tyr^323^ could play a role in the modulation of the Vav activity at the leading edge [46]. Therefore, Syk-Tyr^323^ as well as Syk-Tyr^348^ may be required for the spatio-temporal coordination of Vav-mediated and PI3K p110δ-mediated signaling pathways, a prerequisite for efficient β_2_ integrin-mediated migration of PMN.

In the present study, we demonstrate that Syk activity was required for the formation of PI3K products at the leading edge of polarized dHL-60 on immobilized fibrinogen. The enrichment of PI3K products, especially PIP3, is regulated by different feed-forward mechanisms and is essential for the stabilization of cell polarity [3], [37]. The spatio-temporal control of PI3K p110δ activity by Syk may be involved in the amplification of migratory signals and may be critical for the localization of this process at the leading edge (Figure 6). In accordance with our findings, Popa-Nita et al. have shown recently that the stimulation of human PMN with monosodium urate crystals led to the stimulation of class I_A_ PI3K by a mechanism that was dependent on Syk activity and induced the formation of a complex containing the PI3K regulatory subunit p85 and Syk [25]. Although, the molecular mechanism underlying the activation of PI3K and Syk was not addressed in this study it strongly suggests a potential role of Syk-mediated signaling via class I_A_ PI3K in inflammatory diseases, e.g. gout or gouty arthritis [25]. Furthermore, two recent studies have shown that the fMLP-dependent generation of PI3K products was bi-phasic in neutrophil-like differentiated PLB-85 cells [47] and in murine PMN [48]. In the second phase PI3K products were generated by PI3K p110δ. This second phase was dependent on the first phase generated by PI3K p110γ indicating a functional cross talk between Class I_A_ and I_B_ PI3K signaling [48].

In this study, we present evidence that hematopoietic Syk was required for PMN recruitment to wounded tissue and for subsequent wound healing. In accordance with these findings, we reported previously that Syk was required for PMN adhesion and migration in the model of the inflamed cremaster muscle in vivo [10]. Similar in vivo effects characterized by delayed wound closure due to impaired PMN infiltration and reduced myofibroblast differentiation have been described for CD18^−/−^ mice [28]. Accordingly, impaired neovascularization of the granulation tissue has been reported in CD18^−/−^ mice which may imply that the impairment of inflammation-mediated angiogenesis may contribute to poor wound healing in the absence of efficient PMN infiltration [49]. In a human model of wounded skin, PMN have been demonstrated to differentially express genes that are known to be important for the modulation of the inflammatory response of macrophages, T cells and themselves [50]. Interestingly, PMN seem to change the responsiveness to chemotactic and immunoregulatory mediators once they have migrated to skin lesions and have been activated [50]. Furthermore, the up-regulation of different genes in PMN could play a critical role in the mediation and regulation of processes which are critical for wound healing, e.g. the breakdown of fibrin clots and degradation of extracellular matrix, the promotion of angiogenesis and the migration and proliferation of keratinocytes and fibroblasts [50]. Moreover, CD18 has been shown to be critically involved in homing of endothelial progenitor cells (EPC) and in neovascularization after ischemia and reperfusion injury [51]. Two recent studies have demonstrated a role for Syk and its substrate SLP-76 for vascular and lymphatic development and repair, a process which critically depended on EPC function in a cell-autonomous way [52], [53] and on integrin-mediated signaling [8]. Thus, CD18-mediated Syk signaling seems to be of functional importance for the early recruitment of blood-derived cells including e.g. EPC as well as PMN into the inflamed tissue.

A growing body of evidence indicate that PI3K and/or Syk may represent promising targets for the treatment of diverse inflammatory disorders that are triggered by uncontrolled innate and adaptive immune responses [11], [54]. For example, the inhibition of PI3K p110δ led to reduced PMN-mediated lung inflammation in a murine asthma model [55]. In a previous study, we demonstrated that Syk activity was required for efficient PMN recruitment in two different murine models of acute inflammation, i.e. the reverse Arthus reaction and the inflamed cremaster muscle [10]. In accordance with our in vivo findings, a study by Puri et al. revealed a role for PI3K p110δ in mediating PMN-endothelial interactions and PMN extravasation into the inflamed tissue [32]. Moreover, a recent study by Liu et al. demonstrated by means of intravital microscopy that PI3K p110γ and p110δ have temporally distinct roles for leukocyte recruitment into the inflamed cremaster muscle of mice [56]. Leukocyte emigration was impaired in PI3K p110γ-deficient mice in an early response (within the first 90 min) to the CXC chemokines MIP-2 (CXCL2) or KC (CXCL1). In contrast, the pharmacological inhibition of PI3K p110δ had no effect on the early leukocyte recruitment but it diminished late (after 4–5 h) chemokine-induced PMN recruitment into inflamed tissue in vivo [56]. Taken together, we propose a novel role of Syk for the spatio-temporal control of PI3K p110δ activity during β_2_ integrin-mediated migration of PMN which seems to be important for efficient PMN recruitment into the wounded tissue and cutaneous wound closure (Figure 6).

## Supporting Information

Figure S1 Syk-Tyr^323^ was not required for the tranlsocation of Vav, PLCγ-1, PLCγ-2 and Dock2. Confocal microscopy images of dHL-60 cells expressing EGFP-Syk or EGFP-Syk Y323F. Upon stimulation by 100 nM fMLP on immobilized fibrinogen, Vav (A), PLCγ-1 (B), PLCγ-2 (C), or Dock2 (E) were enriched at the lamellipodium of EGFP-Syk as well as of EGFP-Syk Y323F transfectants (arrows and arrow heads). However, PLCγ-2 and EGFP-Syk Y323F colocalization at the leading edge was absent or weak in some lamellipodia (open arrowhead). (F) Quantitative analysis using microscopic images of fixed dHL-60 cells were taken from three (A,B,C) or two (D) independent experiments. Data represent means±SD. Bar = 10 µm.
(3.74 MB TIF)

Movie S1 Time-lapse microscopy of dHL-60 cells transiently expressing EGFP-Syk. Migration was analyzed in IBIDI-µ slides on immobilized fibrinogen in response to a gradient of 10 nM fMLP. Images were recorded at intervals of 30 s. The movie is representative for at least three independent experiments.
(2.40 MB MOV)

Movie S2 Time-lapse microscopy of dHL-60 cells transiently expressing EGFP-Syk Y323F. Migration was analyzed in IBIDI-µ slides on immobilized fibrinogen in response to a gradient of 10 nM fMLP. Images were recorded at intervals of 30 s. The movie is representative for at least three independent experiments.
(2.74 MB MOV)
