# Supplementary figures and images for: Syk-Mediated Translocation of PI3Kδ to the Leading Edge Controls Lamellipodium Formation and Migration of Leukocytes
Source: PLoS One. 2007 Nov 7;2(11):e1132. doi: 10.1371/journal.pone.0001132 (PMC2063580; doi:10.1371/journal.pone.0001132)

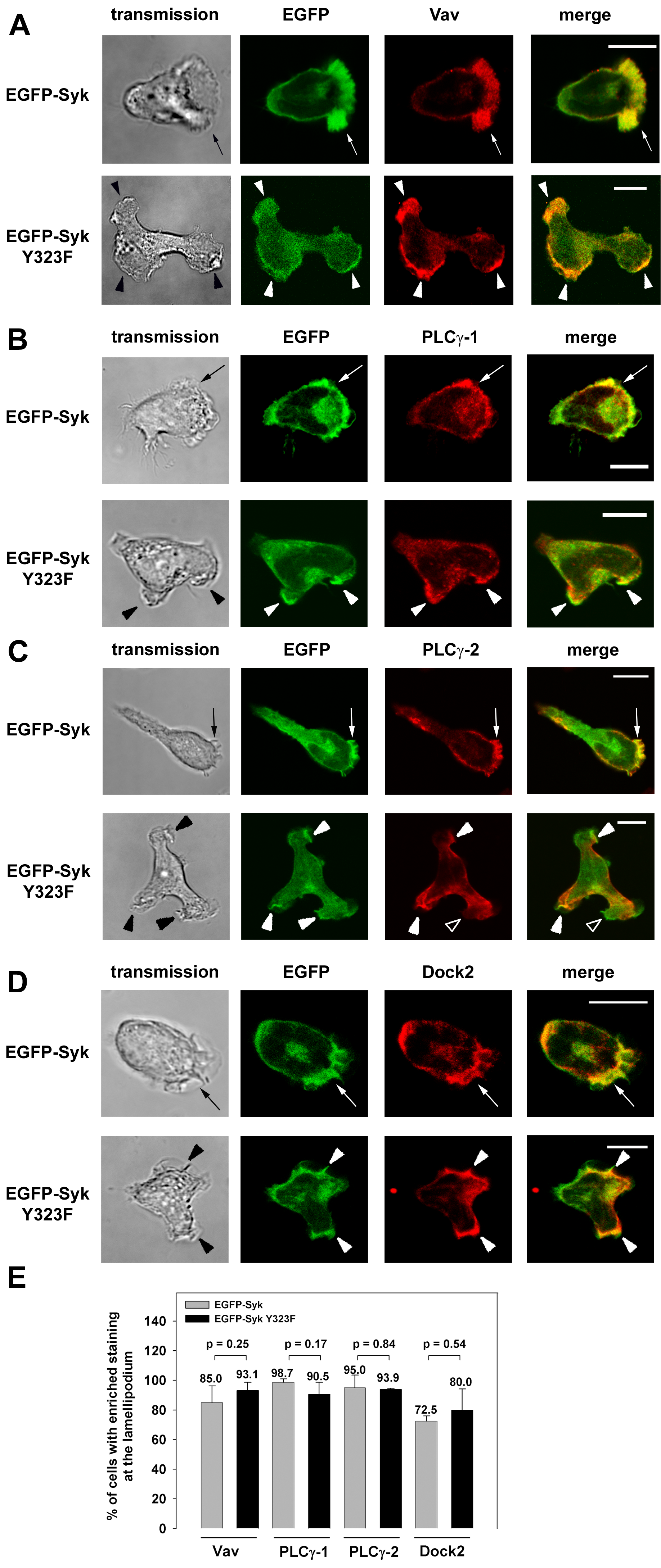

Supplement: Figure S1 — Syk-Tyr323 was not required for the tranlsocation of Vav, PLCγ-1, PLCγ-2 and Dock2. Confocal microscopy images of dHL-60 cells expressing EGFP-Syk or EGFP-Syk Y323F. Upon stimulation by 100 nM fMLP on immobilized fibrinogen, Vav (A), PLCγ-1 (B), PLCγ-2 (C), or Dock2 (E) were enriched at the lamellipodium of EGFP-Syk as well as of EGFP-Syk Y323F transfectants (arrows and arrow heads). However, PLCγ-2 and EGFP-Syk Y323F colocalization at the leading edge was absent or weak in some lamellipodia (open arrowhead). (F) Quantitative analysis using microscopic images of fixed dHL-60 cells were taken from three (A,B,C) or two (D) independent experiments. Data represent means±SD. Bar = 10 µm. (3.74 MB TIF) [file pone.0001132.s001.tif]
